# Appendicular skeletal muscle mass: A more sensitive biomarker of disease severity than BMI in adults with mitochondrial diseases

**DOI:** 10.1371/journal.pone.0219628

**Published:** 2019-07-25

**Authors:** Yue Hou, Zhiying Xie, Xutong Zhao, Yun Yuan, Pan Dou, Zhaoxia Wang

**Affiliations:** 1 Department of Neurology, Peking University First Hospital, Beijing, China; 2 Department of Clinical Nutrition, Peking University First Hospital, Beijing, China; RIKEN Advanced Science Institute, JAPAN

## Abstract

The study aimed to evaluate the body composition of patients with mitochondrial diseases (MD) and correlate it with disease severity. Overall, 89 patients (age ≥ 18 years) with MD were recruited, including 49 with chronic progressive external ophthalmoplegia (CPEO) and 40 with mitochondrial encephalomyopathy with lactate acidosis and stroke-like episodes (MELAS). Body composition, including fat mass index (FMI), fat-free mass index (FFMI), skeletal muscle mass index (SMI), and appendicular skeletal muscle mass index (ASMI), were examined using multifrequency bioelectric impedance analysis. Clinical assessments, including muscle strength, usual gait speed, and disease severity determined by the Newcastle Mitochondrial Disease Adult Scale score (NMDAS), were performed. The comparisons between patients group and age- and gender-matched healthy controls, as well as the correlations between anthropometric measurements, body composition, and disease severity were analyzed. Height, weight, body mass index (BMI), FFMI, SMI, and ASMI were significantly lower in patients with MD than in healthy controls. Notably, low muscle mass was noted in 69.7% (62/89) of MD patients, with 22 patients also presenting with compromised physical performance as indicated by decreased gait speed, resulting in 24.7% satisfied the sarcopenia diagnostic criteria. Disease severity was more negatively correlated with ASMI than it was with height, weight, and BMI. Subgroup analysis showed that in the MELAS subgroup, disease severity was negatively correlated with height, weight, and ASMI; whereas in the CPEO subgroup, it was only negatively correlated with ASMI and SMI. Additionally, ASMI was positively associated with muscle strength. Altogether, compared with BMI, ASMI is a more sensitive biomarker predicting disease severity of MD, both in MELAS and CPEO patients.

## Introduction

Mitochondrial diseases (MD) are a group of clinically and genetically heterogeneous disorders caused by dysfunction of the mitochondrial respiratory chain either due to nuclear deoxyribonucleic acid (DNA) or mitochondrial DNA (mtDNA) defects [1]. Impaired mitochondrial function has a detrimental effect on the cellular energy generating capacity and activities; therefore, tissues with high energy demands such as the nervous system, muscle, and heart are most often affected. While most mitochondrial disorders manifest with different combinations of multiple system involvements, comprising distinct syndromes such as mitochondrial encephalomyopathy with lactic acidosis and stroke-like episodes (MELAS), Kearns-Sayre syndrome (KSS), or myoclonic epilepsy with ragged red fibers (MERRF), some patients present with the predominant involvement of a single organ such as those with chronic progressive external ophthalmoplegia (CPEO) and isolated mitochondrial myopathy (MM) [2,3]. The diagnosis of mitochondrial disease is based on clinical, biochemical, myopathological, and genetic features [4].

The development of biomarkers of MD has significant value for the evaluation and monitoring of disease severity because of the high degree of heterogeneity within phenotypic groups of MD and a lack of disease-modifying treatments with clear clinical benefits currently. The current biomarkers of MD include the Newcastle Mitochondrial Disease Adult Scale (NMDAS), circulating serum markers, imaging markers, and metabolomic markers [5,6]. Additionally, anthropometric measurements such as height, body weight, and body mass index (BMI) have been investigated as potential clinical biomarkers of both mtDNA mutations and nuclear gene mutations because short stature and underweight are common features in patients with these mutations [7–9].

BMI, though is a commonly used indicator, has low sensitivity in accurately reflecting the body compartments and distribution [10–12]. Bioelectrical impedance analysis (BIA), a non-invasive and valid technique to analyze body composition, has been widely used in studying a range of chronic disorders and neuromuscular diseases [13,14,12,15,16]. So far, little is known about the alterations in the body components in patients with mitochondrial defects and sequentially targeted intervention strategies are lacking. Herein, We investigated the body composition in patients with mitochondrial disorders and healthy controls using BIA and performed correlation analysis between body composition, anthropometric measurements, and disease severity.

## Subjects and methods

### Subjects

This was a prospective cross-sectional study. A total of 89 adult patients (age ≥ 18 years; 57 women and 32 men) with MD were recruited from the Department of Neurology at Peking University First Hospital between April 2016 and June 2018, including 49 patients with CPEO (40 sporadic cases with single large-scale mtDNA deletion and 9 autosomal inherited cases with nuclear gene defect *POLG*, *RRM2B*, *Twinkle*, *or TK2*) and 40 patients with MELAS (36 with m.3243A>G, 2 with 8344A>G, 1 with 5541C>T, and 1 with 10158C>T mutations). The patients with CPEO mainly presented with ophthalmoplegia, exercise intolerance, limb weakness, dysarthria, neuropathy, cardiac conduction block. Those with MELAS mainly presented with encephalomyopathic form of stroke-like episodes, epilepsy, cognitive decline, hearing loss, exercise intolerance, and limb weakness. The diagnosis of all patients was confirmed by muscle histopathology and molecular genetic examinations as previous reported. Comprehensive massively parallel sequencing, or polymerase chain reaction-restriction fragment length polymorphism (PCR-RFLP), or long range polymerase chain reaction (LR-PCR) were used to detect mtDNA point variant or mtDNA deletion[17–19]. And targeted next-generation sequencing (NGS) was applied to detect nuclear gene defect[20]. And 178 healthy participants were recruited randomly as controls for a 2:1 match with the patients in terms of age and sex. Patients and controls with neoplasms, connective tissue abnormalities, severe digestive system diseases, inflammation, glucocorticoid therapy history, smoking history, or any other comorbidity were excluded from the study. Face to face interview with open-ended questions was used to evaluate the decision-making capacity. And then written informed consent was obtained from all participants or their legal guardians. This study was approved by the Ethics Committee of Peking University First Hospital.

### Clinical assessments

The clinical assessments of all the patients were evaluated by at least two experienced neurologists, including NMDAS, skeletal muscle strength, and usual gait speed assessments. NMDAS was used to assess the disease severity and involvements of multiple systems [5,21]. Muscle strength was tested bilaterally using the standardized manual muscle testing of Medical Research Council (MRC) Scale, which was converted to a 0–10 point scale in nine muscle groups (neck flexion, shoulder abduction, elbow flexion and extension, hip flexion, knee flexion and extension, and ankle dorsiflexion and plantarflexion) [22,23]. Subsequently, the average score of the muscle strength was calculated. The usual gait speed was evaluated based on walking at the usual pace for a distance of 5 meter [24]. Usual gait speed < 0.8 m/s was established as the cut-off for poor physical performance [25]. It should be noted that in patients with MELAS, muscle strength and usual gait speed were tested during the stable or recovery phases. In addition, all the patients were evaluated by the Gastrointestinal Symptom Rating Scale (GSRS), a self-report questionnaire that includes 15 items regarding gastrointestinal symptoms during the preceding four weeks. The severity of the symptoms is rated on a scale of 0–6, where 0 implies “no complaints” and 6 implies very troublesome symptoms. Patients with GSRS scores > 8, or a score > 2 for any single item, were excluded from the study [26–28].

### Anthropometric measurements and body composition analysis

According to standard procedures [29], height was measured using a wall-mounted stadiometer to the nearest 0.1 cm. Body weight was measured with a digital scale to the nearest 0.1 kg. BMI was defined as weight divided by the square) of the height in meters (kg/m^2^). Body composition was assessed using direct segmental 8-point multifrequency bioelectric impedance analysis (InBody770, InBody Co., Ltd, Seoul, Korea), which had excellent agreement with dual-energy X-ray absorptiometry and magnetic resonance imaging (MRI) [30–32]. The parameters of body composition included fat mass (FM), fat-free mass (FFM), skeletal muscle mass (SM), and appendicular skeletal muscle mass (ASM). ASM was defined as the sum of muscle mass in both the arms and legs. The fat mass index (FMI), fat-free mass index (FFMI), skeletal muscle mass index (SMI), and appendicular skeletal muscle mass index (ASMI) were calculated as FM, FFM, SM, and ASM divided by the square of height (kg/m^2^), respectively [32]. Low muscle mass was defined as ASMI < 7.0 kg/m^2^ in men and < 5.7 kg/m^2^ in women [24,25].

### Statistical analysis

All data were analyzed using SPSS version 20 software (IBM Inc., Armonk, NY). Continuous variables were compared between the two groups using unpaired student’s *t*-test for independent variables with non-equal variances. Comparisons of categorical variables were performed with chi-square test. Generalized linear models were used to adjust for age and sex in testing the differences in the means between the patients with CPEO and MELAS. Two-tailed Spearman correlation analysis was employed for correlation analyses between height, weight, BMI, body composition (ASMI and SMI), and main clinical data (age, age at onset, NMDAS score, muscle strength, and usual gait speed). Two-tailed P values < 0.05 were considered statistically significant.

## Results

### Basic clinical data and physical performance

The mean age of the patients with MD was 30.4 ± 10.3 years, and the mean age at disease onset was 20.4 ± 8.9 years. The mean NMDAS score was 12.4 ± 6.8 and muscle strength score was 9.4 ± 0.7. In the CPEO subgroup (29 women and 20 men; mean age, 32.0 ± 10.9 years), the mean NMDAS score was 9.0 ± 5.8 and muscle strength score was 9.3 ± 0.7. In the MELAS subgroup (28 women and 12 men; mean age, 28.4 ± 9.3 years), the mean NMDAS score was 16.6 ± 5.5 and the average strength score was 9.4 ± 0.5. The patients with MELAS had shorter disease duration than did those with CPEO (8.5 ± 4.5 vs. 11.2 ± 7.6 years, p = 0.040), and the disease severity in MELAS was higher than those in CPEO (p < 0.001). Gait speed < 0.8 m/s was noted in 32.6% (29/89) of MD patients and no significant difference with respect to gait speed was found between the patients with MELAS and those with CPEO.

### Anthropometric measurements and body composition analysis

**Fig 1:**
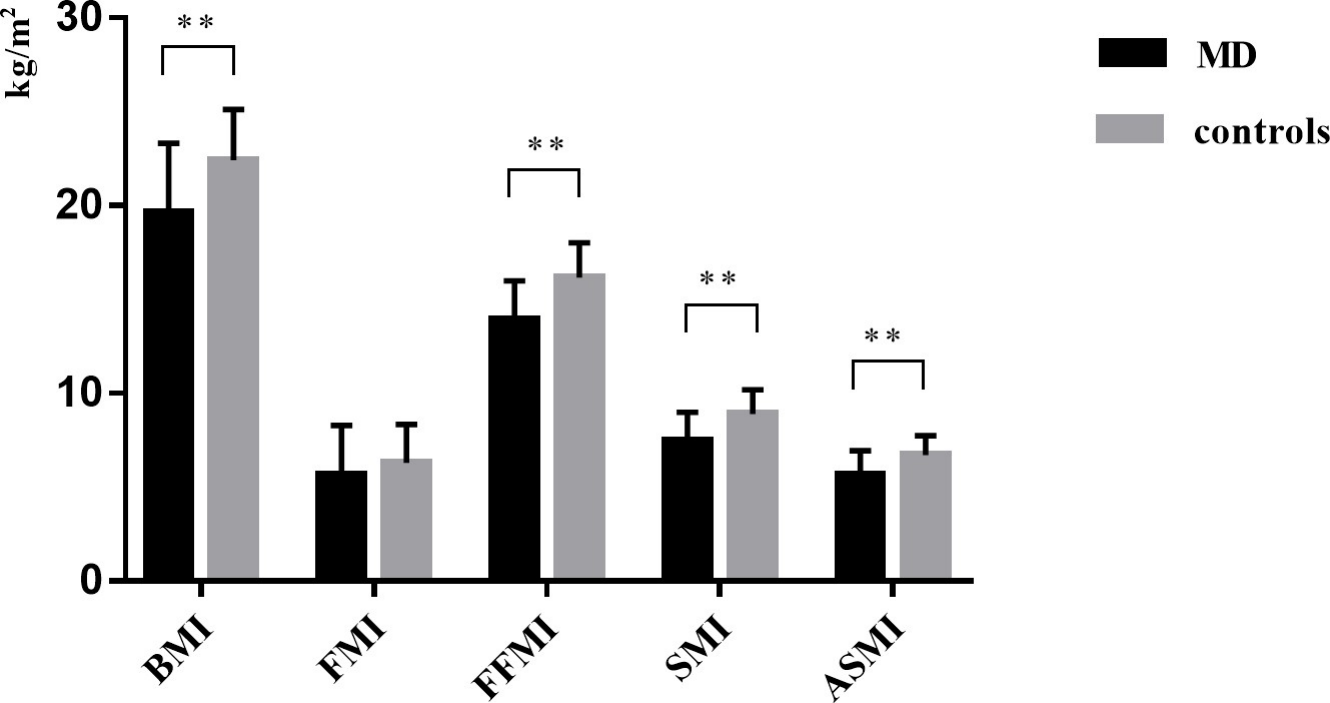
Comparison of BMI, FMI, FFMI, SMI, ASMI between patients with MD and healthy controls. ** statistical significance (p < 0.01). BMI, body mass index; FMI, fat mass index; FFMI, fat-free mass index; SMI, skeletal muscle mass index; ASMI, appendicular skeletal muscle mass index; MD, mitochondrial diseases.

As shown in Table 1, height, weight, and BMI were lower in patients with MD than in healthy controls (p < 0.001). Notably, 34.7% (17/49) of the patients with CPEO and 45.0% (18/40) of the patients with MELAS had BMI < 18.5 kg/m^2^. Body composition analysis revealed that FFMI, SMI, and ASMI were significantly lower in patients with MD than in controls (p < 0.001) (Fig 1). Furthermore, the prevalence of low muscle mass was 69.7% (62/89) in patients with MD and 16.9% (30/178) in controls; 73.7% (42/57) of women with MD had ASMI < 5.7 kg/m^2^ and 62.5% (20/32) of men with MD had ASMI < 7.0 kg/m^2^. When combined with the usual gait speed, 24.7% (22/89) of patients with MD (26.3% of women and 21.9% of men) had low muscle mass as well as impaired gait speed.

**Table 1:** Anthropometric measurements, body composition and usual gait speed in patients with MELAS, CPEO, MD, and healthy controls.

	MELAS (n = 40)	CPEO (n = 49)	MD (n = 89)	Controls (n = 178)
Sex (F/M)	28/12	29/20	57/32	114/64
Age (y)	28.4 ± 9.3	32.0 ± 10.9	30.4 ± 10.3	30.4 ± 10.3
Anthropometric measurements				
Height (cm)	159.9 ± 8.9^&^	163.3 ± 8.1	161.8 ± 8.6^#^	164.9 ± 7.6^&^^#^
Body weight (kg)	48.9 ± 11.7^&^	53.9 ± 12.6*	51.7 ± 12.4^#^	61.2 ± 9.8^&^*^#^
BMI (kg/m^2^)	19.03 ± 3.18^&^	20.11 ± 4.07*	19.63 ± 3.71^#^	22.42 ± 2.69^&^*^#^
Body composition and usual gait speed				
FMI (kg/m^2^)	4.91 ± 2.21^&^^§^	6.28 ± 2.83^§^	5.66 ± 2.65	6.29 ± 2.07^&^
FFMI (kg/m^2^)	14.03 ± 1.74^&^	13.84 ± 2.33*	13.92 ± 2.08^#^	16.16 ± 1.85^&^*^#^
SMI (kg/m^2^)	7.58 ± 1.61^&^	7.37 ± 1.50*	7.46 ± 1.54^#^	8.89 ± 1.30^&^*^#^
Female (kg/m^2^)	7.07 ± 0.91^&^	6.86 ± 1.18*	6.96 ± 1.05^#^	8.22 ± 0.99^&^*^#^
Male (kg/m^2^)	8.77 ± 2.23^&^	8.10 ± 1.63*	8.35 ± 1.87^#^	10.09 ± 0.83^&^*^#^
ASMI (kg/m^2^)	5.63 ± 1.43^&^	5.66 ± 1.21*	5.65 ± 1.30^#^	6.72 ± 1.01^&^*^#^
Female (kg/m^2^)	5.16 ± 0.77^&^	5.13 ± 0.85*	5.14 ± 0.81^#^	6.16 ± 0.73^&^*^#^
Male (kg/m^2^)	6.74 ± 1.96^&^	6.44 ± 1.25*	6.55 ± 1.53^#^	7.71 ± 0.61^&^*^#^
% reduced ASMI	72.5 (29) ^&^	67.4 (33) *	69.7 (62) ^#^	16.9 (30) ^&^*^#^
Female (%)	75.0 (21) ^&^	72.4 (21) *	73.7 (42) ^#^	20.2 (23) ^&^*^#^
Male (%)	66.7 (8) ^&^	60.0 (12) *	62.5 (20) ^#^	10.9 (7) ^&^*^#^
UGS (m/s)	0.92 ± 0.21	0.90 ± 0.17	0.91 ± 0.19	
% reduced UGS	35.0 (14)	30.6 (15)	32.6 (29)	
% reduced ASMI and UGS	25.0 (10)	24.5 (12)	24.7 (22)	
Female (%)	25.0 (7)	27.6 (8)	26.3 (15)	
Male (%)	25.0 (3)	20.0 (4)	21.9 (7)	

Data shown as mean ± standard deviation or percentage (number); MELAS, encephalomyopathy with lactate acidosis and stroke-like episodes; CPEO, chronic progressive external ophthalmoplegia; MD, mitochondrial diseases; F, female; M, male; y, years; BMI, body mass index; FMI, fat mass index; FFMI, fat-free mass index; SMI, skeletal muscle mass index; ASMI, appendicular skeletal muscle mass index; UGS, usual gait speed. Reduced ASMI indicates ASMI < 5.7 kg/m^2^ in women and < 7.0 kg/m^2^ in men. Reduced UGS indicates usual gait speed < 0.8 m/s.

^#^ statistical significance between patients with MD and controls (p < 0.05).

^&^ statistical significance between patients with MELAS and controls (p < 0.05).

* statistical significance between patients with CPEO and controls (p < 0.05).

^§^ statistical significance between patients with MELAS and patients with CPEO (p < 0.05).

Subgroup analysis revealed that, on an average, patients with MELAS were 5.0 cm shorter than healthy controls (p = 0.002), while no significant difference was found between those with CPEO and controls. The patients with MELAS had a weak tendency to be shorter and thinner than those with CPEO, although there was no significant difference (p>0.05). FMI was lower in patients with MELAS when compared with those with CPEO and controls (p = 0.012 and p = 0.001, respectively) and there was no significant difference between patients with CPEO and controls.

### Correlations of body composition and clinical manifestation

The results of the correlation analysis in patients with MD were summarized in Table 2. Spearman’s correlation analysis in patients with MD demonstrated a negative correlation of disease severity with ASMI (r = -0.325, p = 0.002), which was stronger than the correlations between disease severity and height (r = -0.249, p = 0.019), weight (r = -0.298, p = 0.005), and BMI (r = -0.249, p = 0.018). Subgroup analysis showed that while in the MELAS subgroup, NMDAS score had negative correlations with height (r = -0.445, p = 0.004), weight (r = -0.364, p = 0.021), and ASMI (r = -0.351, p = 0.027), in the CPEO subgroup, it was negatively correlated with only ASMI (r = -0.357, p = 0.012) and SMI (r = -0.468, p = 0.001)(S1 Table). Furthermore, patients with higher ASMI had better muscle strength (r = 0.405, p<0.001). Additionally, weight (r = 0.382, p<0.001), BMI (r = 0.342, p = 0.001) were correlated with the age of onset.

**Table 2:** Correlation coefficients (r) in patients with MD.

Variables	Height (cm)		Weight (kg)		BMI (kg/m^2^)		SMI (kg/m^2^)		ASMI (kg/m^2^)	
	r	p	r	p	r	p	r	p	r	p
Age (y)	0.034	0.750	0.262*	0.013	0.240*	0.023	0.123	0.252	0.153	0.154
Age at onset (y)	0.112	0.297	0.382**	0.000	0.342**	0.001	0.309**	0.003	0.290**	0.006
NMDAS score	-0.249*	0.019	-0.298**	0.005	-0.249*	0.018	-0.280**	0.008	-0.325**	0.002
Muslce strength	0.154	0.151	0.270*	0.010	0.270*	0.010	0.469**	0.000	0.405**	0.000
UGS (m/s)	0.186	0.081	0.190	0.075	0.121	0.259	0.278**	0.008	0.252*	0.017

BMI, body mass index; SMI, skeletal muscle mass index; ASMI, appendicular skeletal muscle mass index; y, years; NMDAS, Newcastle Mitochondrial Disease Adult Scale; UGS, usual gait speed.

^*^ statistical significance (p < 0.05).

^**^statistical significance (p < 0.01).

## Discussion

To the best of our knowledge, our study is the first to perform anthropometric measurements and comprehensive body composition analyses in a large cohort of patients with MD, although body composition analysis had been utilized as outcome variables in several small sample-size clinical studies previously [33–35]. Furthermore, we also performed correlation analysis between the various anthropometric measurements, body composition, and disease severity in patients with MD.

MD encompasses a wide range of phenotypical subgroups. In this study, two common adult MD subgroups, including CPEO and MELAS, were enrolled. CPEO caused by single large-scale mtDNA deletion or nuclear gene mutations mainly presents with gradually progressive ptosis and ophthalmoparesis [36]. MELAS has more multisystem manifestations such as stroke-like episodes, seizures, short stature, hearing loss, and diabetes mellitus, in which 3243A>G is responsible for > 80% of the cases [37]. Therefore, CPEO and MELAS can be regarded as representatives of oligo- and multi-system involvements in MD, respectively. Additionally, the genotypes of our patient cohort included mtDNA point mutations, mtDNA single rearrangement, and nuclear gene mutations. Therefore, the present cohort of patients is a representative population of adult patients with MD.

Our findings support those of previous studies that indicated that height, weight, and BMI were significantly lower in patients with MD when compared to healthy peers [38,39,8,40,21]. Further subgroup analyses revealed that patients with MELAS had higher disease severity and a tendency to be shorter and thinner than those with CPEO, which were consistent with previous study[8]. It is reasonable to infer that since MELAS generally affects more systems than does CPEO, multiple factors including growth hormone deficiency, hypothyroidism, and gastrointestinal problems are more prominent in patients with MELAS than those with CPEO [41–43] and they contribute to the failure to thrive and severe nutritional deterioration in patients with MELAS.

A unique finding of our study was that FFMI, SMI, and ASMI were significantly lower in patients with MD when compared with healthy controls. Notably, low muscle mass was observed in 69.7% (62/89) of patients and of these 62 patients, 22 also had poor physical performance[25]. Loss of muscle mass may result in functional impairments eventually. More evidence revealed that mitochondrial dysfunction plays a key role in the pathogenesis of muscle loss [44–46]. Previous studies have reported that abnormal electron transport chain was observed in aging muscles with fiber atrophy [47–49], and induced mtDNA mutation could directly cause muscle wasting [50,46]. Shah et al. reported that mtDNA deletion correlated positively with lean body mass [51]. And people with a high degree of sarcopenia exhibited severe mitochondrial abnormalities [52]. However, muscle loss has not been noticed or highlighted in primary mitochondrial disorders previously, although sarcopenia has been well studied in other secondary mitochondrial dysfunctions such as chronic obstructive pulmonary disease and chronic kidney and liver diseases [53–56]. We propose that both CPEO and MELAS, either caused by mtDNA deletions or point mutations, are involved in mitochondrial defects which may lead to muscle involvement and accelerate the process of muscle mass loss. The recognition of muslce loss in patients with MD is of great significance because there is no effective treatment for MD till date and reversing or preventing muscle loss is an essential supportive measure [57]. Future longitudinal studies on the impact of muscle loss on the prognosis of MD and the precise molecular mechanisms underlying muslce loss in the muscles of patients with MD are needed.

We analyzed the associations between the anthropometric parameters, body composition, and disease severity of MD. Many biomarkers have been previously examined for early diagnostic purposes and predicting the prognosis of MD [6]. A recent study by Boal et al. reported that the height in adult patients with MD can reflect the disease severity [8]. The present study demonstrated that NMDAS score was negatively correlated with ASMI, SMI, height, weight, and BMI in the patient population; in the CPEO subgroup, however, NMDAS score was not correlated with height, weight, or BMI, but was negatively correlated with ASMI and SMI. Additionally, ASMI, in comparison with SMI, had relatively closer association with disease severity in CPEO and MELAS, which indicating muscular of limbs were mostly affected. Therefore, ASMI could be more sensitive biomarkers of disease severity than BMI in patients with MD, including those with CPEO and MELAS. The muscle is the main system involved in CPEO, while multiple systems are affected in MELAS. The different degree of muscle involvement in CPEO and MELAS may contribute to the stronger relationship between appendicular skeletal muscle mass and disease burden in patients with CPEO than in MELAS.

Considering the feasibility, low cost, and repeatability of BIA, appendicular skeletal muscle mass is a promising clinical biomarker worthy of investigation in future longitudinal studies for monitoring disease progression in MD. Assessments of the body composition and muscle mass can also guide individualized dietary and physical therapy strategies to maintain muscle performance and, more importantly, improve the oxidative function [58,59,9]. We recommend the use of BIA in routine evaluation of patients with MD.

This study has some limitations. First, this was a cross-sectional, single-center study that only included patients with MELAS and CPEO; longitudinal follow-up studies at multiple centers are needed to verify the correlations between body composition and disease progression in patients with MD. Second, the association between muscle mutation load and muscle mass index was not assessed in the present study due to incomplete genetic data.

In conclusion, muslce loss is relatively common in patients with mitochondrial diseases. Compared with BMI, skeletal muscle mass is a more sensitive biomarker for predicting the disease severity of mitochondrial disorders, both in patients with MELAS and CPEO. We recommend the evaluation of body composition in the nutritional assessment and follow-up of patients with mitochondrial disorders.

## Supporting information

S1 Checklist PLOS One Clinical Studies Checklist-PONE-D-19-07905. (DOC)

S1 Table Correlation coefficients (r) in patients with CPEO and those with MELAS. Data shown as correlation coefficient (significance); BMI, body mass index; SMI, skeletal muscle mass index; ASMI, appendicular skeletal muscle mass index; CPEO, chronic progressive external ophthalmoplegia; MELAS, encephalomyopathy with lactate acidosis and stroke-like episodes; y, years; NMDAS, Newcastle Mitochondrial Disease Adult Scale; UGS, usual gait speed. * statistical significance (p < 0.05). ** statistical significance (p < 0.01).
(DOC)

S1 File Approval document. (JPG)

S2 File Approval document English translation. (DOC)

S3 File Approval document program change application. (PDF)

S4 File Approval document program change application English translation. (DOC)
