# Supplementary material for: Appendicular skeletal muscle mass: A more sensitive biomarker of disease severity than BMI in adults with mitochondrial diseases
Source: PLoS One. 2019 Jul 25;14(7):e0219628. doi: 10.1371/journal.pone.0219628 (PMC6657836; doi:10.1371/journal.pone.0219628)
Supplement: S1 Table — Data shown as correlation coefficient (significance); BMI, body mass index; SMI, skeletal muscle mass index; ASMI, appendicular skeletal muscle mass index; CPEO, chronic progressive external ophthalmoplegia; MELAS, encephalomyopathy with lactate acidosis and stroke-like episodes; y, years; NMDAS, Newcastle Mitochondrial Disease Adult Scale; UGS, usual gait speed. * statistical significance (p < 0.05). ** statistical significance (p < 0.01). (DOC) [file pone.0219628.s002.doc]

**S1 Table.** Correlation coefficients (r) in patients with CPEO and those with MELAS.

| Variables | Height (cm) | | Weight (kg) | | BMI (kg/m^2^) | | SMI (kg/m^2^) | | ASMI (kg/m^2^) | |
| --- | --- | --- | --- | --- | --- | --- | --- | --- | --- | --- |
|  | CPEO | MELAS | CPEO | MELAS | CPEO | MELAS | CPEO | MELAS | CPEO | MELAS |
| Age (y) | 0.083  (0.571) | -0.095  (0.560) | **0.385^**^**  **(0.006)** | -0.120  (0.462) | **0.367^*^**  **(0.010)** | -0.154  (0.344) | 0.090  (0.539) | 0.106  (0.515) | 0.120  (0.410) | 0.110  (0.500) |
| Age at onset (y) | 0.056  (0.704) | 0.149  (0.358) | **0.413^**^**  **(0.003)** | 0.279  (0.081) | **0.391^**^**  **(0.005)** | 0.204  (0.207) | **0.353^*^**  **(0.013)** | 0.271  (0.091) | 0.281  (0.051) | **0.313^*^**  **(0.049)** |
| NMDAS score | 0.004  (0.980） | **-0.445^**^**  **(0.004)** | -0.196  (0.177) | **-0.364^*^**  **(0.021)** | -0.244  (0.091) | -0.263  (0.101) | **-0.468^**^**  **(0.001)** | -0.258  (0.108) | **-0.357^*^**  **(0.012)** | **-0.351^*^**  **(0.027)** |
| Muslce strength | 0.108  (0.459) | 0.242  (0.132) | **0.306^*^**  **(0.034)** | 0.295  (0.064) | **0.311^*^**  **(0.030)** | 0.170  (0.294) | **0.614^**^**  **(0.000)** | 0.258  (0.108) | **0.528^**^**  **(0.000)** | 0.218  (0.177) |
| UGS (m/s) | 0.160  (0.271) | 0.228  (0.157) | 0.201  (0.165) | 0.206  (0.203) | 0.200  (0.168) | 0.031  (0.847) | **0.446^**^**  **(0.001)** | 0.112  (0.493) | **0.406^**^**  **(0.004)** | 0.105  (0.519) |

Data shown as correlation coefficient (significance); BMI, body mass index; SMI, skeletal muscle mass index; ASMI, appendicular skeletal muscle mass index; CPEO, chronic progressive external ophthalmoplegia; MELAS, encephalomyopathy with lactate acidosis and stroke-like episodes; y, years; NMDAS, Newcastle Mitochondrial Disease Adult Scale; UGS, usual gait speed.

^*^ statistical significance (p < 0.05).

^**^ statistical significance (p < 0.01).
