# Supplementary figures and images for: Appendicular skeletal muscle mass: A more sensitive biomarker of disease severity than BMI in adults with mitochondrial diseases
Source: PLoS One. 2019 Jul 25;14(7):e0219628. doi: 10.1371/journal.pone.0219628 (PMC6657836; doi:10.1371/journal.pone.0219628)

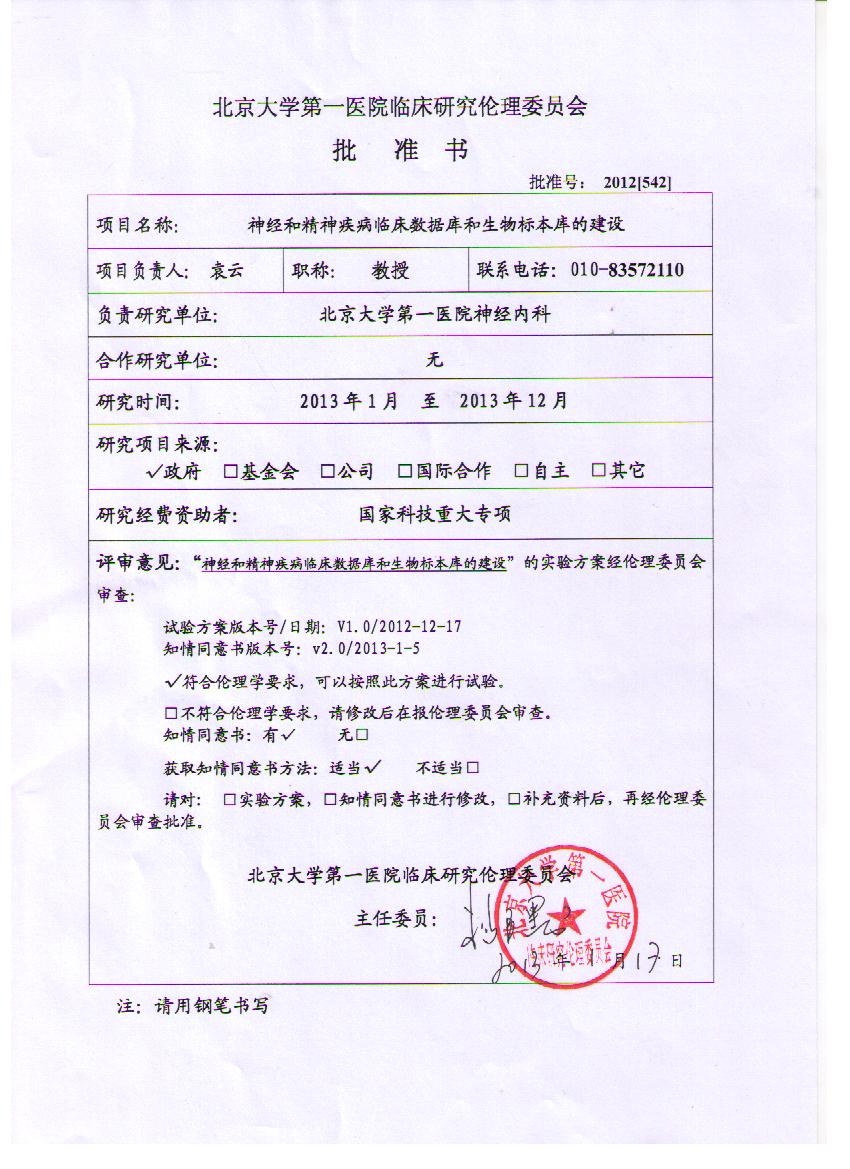

Supplement: S1 File — (JPG) [file pone.0219628.s003.jpg]
