# Supplementary material for: Appendicular skeletal muscle mass: A more sensitive biomarker of disease severity than BMI in adults with mitochondrial diseases
Source: PLoS One. 2019 Jul 25;14(7):e0219628. doi: 10.1371/journal.pone.0219628 (PMC6657836; doi:10.1371/journal.pone.0219628)
Supplement: S2 File — (DOC) [file pone.0219628.s004.doc]

Human Research Ethics Committee of Peking University First Hospital

Approval document

Approval No.2012[542]

| Project Name: Clinical database and biological sample library of neurological and psychiatric diseases | | |
| --- | --- | --- |
| Person in charge: Yun Yuan | Title: Professor | Phone: 010-83572110 |
| Research institute in charge: Department of Neurology, Peking University First Hospital | | |
| Research institute in cooperation: None | | |
| Research time: Jan 2013 – Dec 2013 | | |
| Sources of research:  √ Government Ο Fund Ο Company Ο International Ο Independent Ο Others | | |
| Funder of research: National Science and Technology Major Project | | |
| Review opinion: The methods of *Clinical database and biological sample library of neurological and psychiatric diseases*  Trial version/date: V1.0/2012-12-17  Consent version: v2.0/2013-1-5  √ Comply with ethical requirements, approve to conduct the research according to the methods  Ο Not comply with ethical requirements, reapply for approval of human research ethics committee  Consent: Yes √ No Ο  Methods of obtaining consent: Appropriate √ Inappropriate Ο    Human Research Ethics Committee of Peking University First Hospital  Chairman: Yang Yingmo  2013-1-17 | | |
