# Supplementary material for: Appendicular skeletal muscle mass: A more sensitive biomarker of disease severity than BMI in adults with mitochondrial diseases
Source: PLoS One. 2019 Jul 25;14(7):e0219628. doi: 10.1371/journal.pone.0219628 (PMC6657836; doi:10.1371/journal.pone.0219628)
Supplement: S3 File — (PDF) [file pone.0219628.s005.pdf]

2015.3.17 递交

2015.3 三十五次会议

北京大学第一医院临床研究伦理审查

方案变更申请书

项目名称：神经和精神疾病临床数据库和生物标本库的建设	
方案变更申请日期：2015 年 3 月 17 日	原项目伦理批准号：2012[542]
**A 主要研究者信息**	
科室：神经内科	姓名：袁云
职称：主任医师	联系电话：010-83572462
电子信箱：yuanyun2002@sohu.com	传真：010-66551107
指定联系人　姓名：王朝霞<br>电子信箱：drwangzx@163.com	手机：13681495953<br>王青青 15501263779 83572588
**B 方案变更内容（请另附页具体说明变更原因和变更内容）**	
☐ 试验方案版本变更<br>☐ 知情同意书版本变更<br>☐ 更改、扩大或新增研究内容<br>☐ 新增合作中心/单位<br>☐ 关闭合作中心/单位<br>√ 延长研究时间<br>☐ 其他____________	
**C 方案变更信息（无变化的部分可不填）**	
试验方案版本号：	知情同意书版本号：
拟研究时间：　2015 年 3 月　至 2018 年 3 月	
研究项目来源：☐政府　☐基金会　☐公司　☐国际组织　☐其他：________<br>来源名称：	
资助者类型：　☐政府　☐基金会　☐公司　☐国际组织　☐其他：________	
资助者名称：	
资助者联系人：	
联系方式：	

递交审查资料：

☐项目简介　☐试验方案（注明版本号及日期）

☐知情同意书（注明版本号及日期）　☐其他资料________________

包括：试验用品安全性资料、生产企业资质证明、试验用品提供者的资质证明

保密说明：

D 签字

主要研究者声明

我保证以上信息真实准确，并负责该项目全过程中的质量保证，承诺该项目数据真实可靠，操作规范，符合涉及人的研究伦理要求。如有失实，愿意承担相关责任。

主要研究者：[illegible]　日期：

填表人：[illegible]　日期：2015.3.17

E 秘书组建议处理方式

秘书组审查，建议审查方式：

☐ 审查

☑ 备案

签字：[illegible]　日期：2015.3.18

F 主任委员意见

同意

签字：[illegible]　日期：2.15.3.25

北京大学第一医院 [illegible]

方案变更说明

我科目前承担国家科技重大专项---神经和精神疾病临床数据库和生物标本库的建设，该项目的目的是建设我国神经与精神性重大疾病临床综合资源的数据库，从而提供新药研发所需要的重大疾病临床信息及相关病例资源，为具有我国自主知识产权的创新性药物的研发与评价提供统一共性平台和有力技术支撑。

就目前收集的临床数据与生物标本远不够用于疾病与创新性药物的研究，为了建设该生物标本库并将该项目可持续发展，我科提出延期申请，拟研究时间改为 2015 年 3 月至 2018 年 3 月。我们将收集更多的临床数据和生物标本，为将来的临床以及基础研究提供重要的支撑。
