# Supplementary material for: Appendicular skeletal muscle mass: A more sensitive biomarker of disease severity than BMI in adults with mitochondrial diseases
Source: PLoS One. 2019 Jul 25;14(7):e0219628. doi: 10.1371/journal.pone.0219628 (PMC6657836; doi:10.1371/journal.pone.0219628)
Supplement: S4 File — (DOC) [file pone.0219628.s006.doc]

Human Research Ethics Committee of Peking University First Hospital

Program change application

Approval No.2012[542]

| Project Name: Clinical database and biological sample library of neurological and psychiatric diseases | |
| --- | --- |
| Program change application date: 2015-3-17 | Original approval No.2012[542] |
|  | |
| **A Primary investigator information** | |
| Department: Neurology | Name: Yun Yuan |
| Title: Professor | Phone: 010-83572462 |
| E-mail: [yuanyun2002@sohu.com](mailto:yuanyun2002@sohu.com) | Fax: 010-66551107 |
| Designated contact:  Name: Zhaoxia Wang Phone: 13681495953 E-mail: drwangzx@163.com | |
| **B Program change content** | |
| - Change research scheme version - Change informed consent version - Change, enlarge or add research content - Add cooperating center/ organization - Close cooperating center/ organization   **√** Extend research time   - Others______ | |
| **C Program change information** (the part that does not change can be left blank) | |
| Research scheme version No.: | Informed consent version No.: |
| Research time: from 2015-3 to 2018-03 | |
| Sources of research: 🞏 Government 🞏 Fund 🞏 Company 🞏 International organization 🞏 Others_____  Name of the source: | |
| Funder type: 🞏 Government 🞏 Fund 🞏 Company 🞏 International organization 🞏 Others_____ | |
| Funder name: | |
| Funder contact: | |
| Contact information: | |
| Review materials:  🞏 Project introduction 🞏 Research scheme (version and date) 🞏 Informed consent (version and date) 🞏 Others____ | |
| Confidential statement: | |
| **D Signature** | |
| Primary investigator statement  I guarantee that the above information is true and accurate, and I responsible for the quality assurance throughout the project. I promise that the project date is authentic and reliable, and the operation is standardized, and the project is in line with research ethical requirement. If it is not true, I’m willing to take responsibility.  Primary investigator: Yun Yuan Date: | |
| Preparer: Qingqing Wang Date: 2015-3-17 | |
| **E Review team comments:** | |
| Recommended review method  🞏 Review  🞏 Filing  Signature: Ronghui Yu Date: 2015-3-18 | |
| **F Chairman comments** | |
| Approve  Signature: Yining Huang Date: 2015-3-25 | |
